# The Effect of Normoxic and Hypoxic U-87 Glioblastoma Paracrine Secretion on the Modulation of Brain Endothelial Cells

**DOI:** 10.3390/cells11020276

**Published:** 2022-01-14

**Authors:** Mariam Rado, Brian Flepisi, David Fisher

**Affiliations:** 1 Medical Bioscience Department, Faculty of Natural Sciences, University of the Western Cape, Robert Sobukwe Road, Bellville 7535, South Africa; 3580480@myuwc.ac.za; 2 Department of Pharmacology, Faculty of Health Sciences, University of Pretoria, 9 Bophelo Road, Pretoria 0002, South Africa; brian.flepisi@up.ac.za

**Keywords:** glioblastoma multiforme, U-87 cells, tumour secretome, paracrine effects, tumour hypoxia, brain endothelial cells, bEnd.3 cells, blood-brain barrier

## Abstract

Background: Glioblastoma multiforme (GBM) is a highly invasive brain tumour, characterized by its ability to secrete factors promoting its virulence. Brain endothelial cells (BECs) in the GBM environment are physiologically modulated. The present study investigated the modulatory effects of normoxically and hypoxically induced glioblastoma U-87 cell secretions on BECs. Methods: Conditioned media (CM) were derived by cultivating U-87 cells under hypoxic incubation (5% O_2_) and normoxic incubation (21% O_2_). Treated bEnd.3 cells were evaluated for mitochondrial dehydrogenase activity, mitochondrial membrane potential (ΔΨm), ATP production, transendothelial electrical resistance (TEER), and endothelial tight-junction (ETJ) gene expression over 96 h. Results: The coculture of bEnd.3 cells with U-87 cells, or exposure to either hypoxic or normoxic U-87CM, was associated with low cellular viability. The ΔΨm in bEnd.3 cells was hyperpolarized after hypoxic U-87CM treatment (*p* < 0.0001). However, normoxic U-87CM did not affect the state of ΔΨm. BEC ATP levels were reduced after being cocultured with U-87 cells, or with hypoxic and normoxic CM (*p* < 0.05). Suppressed mitochondrial activity in bEnd.3 cells was associated with increased transendothelial permeability, while bEnd.3 cells significantly increased the gene expression levels of ETJs (*p* < 0.05) when treated with U-87CM. Conclusions: Hypoxic and normoxic glioblastoma paracrine factors differentially suppressed mitochondrial activity in BECs, increasing the BECs’ barrier permeability.

## 1. Introduction

Brain endothelial cells (BECs) are the main functional and regulatory components of the blood-brain barrier (BBB). They are characterized by the presence of continuous apicolateral zones of structural proteins called tight junctions (TJs) which link the BECs together, thus significantly limiting the paracellular flux of solutes and the movement of blood-borne cells into the brain. The regulatory function of BECs is modulated by both astrocytes and pericytes, the former enveloping more than 99% of the external surface of the brain’s capillary endothelium with their “endfoot” processes, which provides regulatory feedback from the neuronal environment to the endothelium of brain capillaries [1]. TJs between the cerebral endothelial paracellular spaces have been reported to be more rigorous than in other tight epithelia in the body [2]. Nevertheless, the BBB was reported to be structurally and functionally disrupted by the fast-growing and aggressive brain tumour called glioblastoma multiforme (GBM), also referred to as a grade IV astrocytoma [3,4]. Furthermore, the GBM local environment causes BECs to develop abnormal phenotypes demonstrating hyperplastic and heterogeneous sizes and shapes [5].

GBM is the most malignant type of brain tumour [6], presenting a high mortality rate despite the therapeutic approaches, including surgery, chemotherapy, and radiotherapy. GBM patients have a median overall survival of approximately 15 months [7] and represent 50% of all malignant, aggressive primary brain tumours in humans [8].

Morphologically, GBM tumours can be differentiated into three zones depending on their proximity to the blood vessels (source of O_2_), including the perivascular zone (GBM surrounded by blood vessels), the hypoxic zone (in the core of the GBM tumour tissue), and the invasive zone (that area of the tumour surrounded the vascular zone) [9]. GBM cells in these zones are functionally different, largely based on the O_2_ availability. GBM in the perivascular zone causes an abnormally high rate of angiogenesis due to the increased secretion of paracrine factors that cause disorganized blood-vessel formation. In contrast, GBM cells in the hypoxic zone have a low proliferation level with a high expression of hypoxia-inducible factors (HIF) which modulate cellular homeostasis. In the invasive zone, hypoxic GBM cells infiltrate the surrounding tissue toward the blood vessels to take advantage of nutrition and O_2_ availability, and also migrate to other sites in the brain. During this process, GBM cells interact with brain stromal cells, such as astrocytes and endothelial cells [10,11]. Factors expressed in endothelial cells (bradykinin, EphrinB2, and interleukin (IL-8)), and in GBM cells (EGFRvIII and MDGI) are thought to be implicated in the chemotaxis of migratory GBM cells across the brain endothelial cells [12]. Clinically, the main problem of GBM is the formation of oedema and the increase of the intracerebral pressure due to the disruption of the BBB at the level of the brain capillary endothelial cell [3,13].

The interaction between GBM cells and the surrounding stromal cells (particularly BECs) is crucial in developing the tumour environment and for tumour progression [14,15]. Both in vitro and in vivo studies have demonstrated that GBM cells secrete paracrine factors [16]. This interaction of the cellular components in the GBM microenvironment is mediated by the secretion of various factors from GBM [16,17], which have autocrine or paracrine effects [18,19]. Invasive cancer cells are characterized by secreted factors that increase their malignancy. These factors are thought to facilitate invasive events, such as the degradation of extracellular matrix (ECM) components, cell detachment, and migration through the basement membrane. Glioblastoma is reported to secrete various types of proteins. In a comparative study to quantify the proteins in the conditioned media of three glioblastoma cell lines (LN18, U118, and U-87), the number of proteins in the U-87 conditioned media was significantly higher than the other cell lines [20]. Glioblastoma releases extracellular vesicles (EVs), carrying molecules such as proteins and microRNAs (miRNAs), vascular growth factors, and IL-6,8, which play a role in inducing BBB breakdown. [16,21]

Although the GBM-induced pathological features in brain endothelial cell morphology have been extensively reported [22,23,24,25], it is still unclear by which mechanisms GBM influences the metabolism of BECs. As it is well known that fast-growing and aggressive tumours outpace tumour angiogenesis and develop various intratumour zones of O_2_ deprivation (hypoxia) [26], the current study aimed to evaluate whether glioblastoma U87 cells or their secretome (supernatant) produced under hypoxic (5% O_2_) and normoxic (21% O_2_) conditions would differentially modulate the metabolism in brain endothelial cells, particularly with respect to mitochondrial activity. Using a brain endothelial cell line (bEnd.3 cells), which is well described in the literature [27], the current study investigated the effects of coculturing glioblastoma cells (U-87 cells), and treatment with selected concentrations of their supernatant-derived conditioned media on bEnd.3 cells’ mitochondrial activity (dehydrogenase activity, mitochondrial membrane potential, and ATP production), and on the permeability across confluent monolayers of bEnd.3 cells (transendothelial electrical resistance (TEER)).

## 2. Materials and Methods

### 2.1. Cell Culture and Supernatant Collection

The murine brain microvascular endothelial cell line (bEnd.3 ATCC^®^ CRL-2299, Gaithersburg, MD, USA) and the invasive human glioblastoma cell line (U-87 MG, ATCC HTB-14, 10801 University Boulevard, Manassas, VA, USA) were cultured in complete Dulbecco’s Modified Eagle Medium ((DMEM), Gibco, No. 22320022, 8717 Grovemont Cir, Gaithersburg, MD, USA), supplemented with 10% fetal bovine serum ((FBS), Biowest, No. 10493-106, 2 Rue du Vieux Bourg, Nuaillé, France), and 100 U/mL penicillin/streptomycin (Gibco, No. 15070063) (Complete DMEM)). TrypLE™ Express Enzyme (Thermo Fisher Scientific, No. A1285901, 168 Third Avenue, Waltham, MA, USA) was used for harvesting the cells.

The U-87 cells’ supernatant was collected to prepare the U-87 conditioned medium (U-87CM), as follows: U-87 cells were grown in 75 cm^2^ culture flasks (1 × 10^5^/flask) in a normal, humidified 5% CO_2_ incubator at 37 °C until they reached 50% confluence; then, the spent growth media were replaced with a fresh complete DMEM. Cells were further incubated either under hypoxic (5% O_2_) or normoxic (21% O_2_) conditions. The incubation under hypoxic conditions was performed by placing the tissue culture flasks in a sterilized modular incubator hypoxia chamber (MIC 101; Billups–Rothenberg, Inc., Sorrento Valley Blvd, San Diego, CA, USA). The hypoxia chamber is provided with a Greisinger oxygen meter with a sensor (GOX 100-0-CO, No. 600437), which allows for the measurement of O_2_ levels during the incubation time. After 48 h of incubation in hypoxic or normoxic conditions, the supernatant was collected in ice-cooled centrifuged tubes, centrifuged at 3500 rpm for 5 min at 4 °C, and then filtered with a GVS filter (0.20 µm) (Bio-Smart Scientific, Park Edge Mews, Edgemead, Link Way, Edgemead, Cape Town, South Africa). The supernatants were collected and aliquoted in 2–5 mL cryovials and stored at −80 °C.

### 2.2. bEnd.3 Cells Exposure to U-87 Conditioned Media (CM)

The collected U-87 supernatants were thawed at room temperature (21 °C) and added to fresh complete DMEM at concentrations of 20%, 40%, and 75%. This was subsequently referred to as U-87 conditioned media (U-87CM). The bEnd.3 monoculture cells were seeded at various seeding densities, depending on the assay to be conducted and incubated, in a 5% CO_2_ incubator at 37 °C for 24 h to allow the cells to attach. Following 24 h incubation, the spent growth media were removed, cells were then exposed to either 20%, 40%, or 75% U-87CM for 24, 48, 72, and 96 h. All media were replaced daily to ensure the continuity of the concentrations used, and to maintain sufficient metabolic substrates for the normal functioning of the cell cultures.

### 2.3. Experimental Design

**Figure 1:**
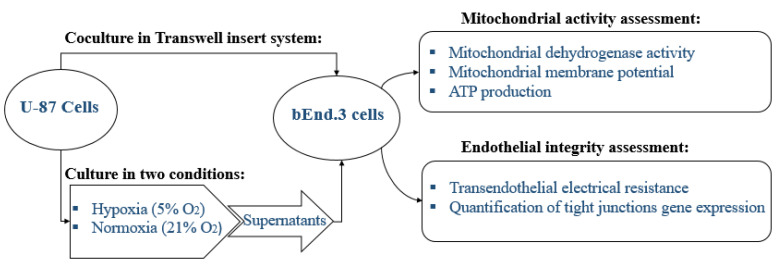
A schematic diagram of the research design to study the effect of the paracrine factors secreted by U-87 cells on bEnd.3 cells.

The study was designed to study the physiological changes in brain endothelial cells bEnd.3 under the influence of U-87 cells. The experiments were carried out in triplicate as a minimum (*n* = 3) and duplicated to ensure repeatability. The effect of normoxic and hypoxic cancerous factors secreted from U-87 cells was compared by treating cultures or monolayers of bEnd.3 cells with selected concentrations of U-87CM or by growing the bEnd.3 cells in a coculture with U-87 cells (Figure 1).

For the in vitro model of the BBB, the bicameral chamber system was used, where the well assumes the abluminal side of the capillary endothelium, while the apical chamber (the insert) assumes the luminal side of the capillary. This allowed for U-87 cells to be cultured in wells, while a three-legged insert (Merck–Millipore, PIHA01250, 6 Hatters Ln, Watford, UK) was used for growing the brain endothelial cell monolayers. These three-legged inserts also facilitated the movement of inserts between varying treatment conditions, e.g., normoxic versus hypoxic conditions.

To ensure the U-87CM had sufficient metabolic constituents, a minimum of 25% fresh growth media (DMEM) was added to the U-87CM to make up the selected treatment concentrations (20%, 40%, and 75%). Furthermore, the U-87 cell cultures were grown to 50% confluency and exposed for a maximum of 48 h, prior to the collection of supernatants and preparation for experimentation. In addition, media for treating cells were replaced daily to ensure continuity in our treatment process.

In addition, the lower level of O_2_ used in the current study was 5% to avoid compromising the viability of the bEnd.3 cells when cocultured under hypoxic conditions. The current study confirmed that bEnd.3 cell monolayers were indeed sensitive to 5% O_2_, but can recover within 24 h to control levels of permeability.

### 2.4. Experiments

#### 2.4.1. Cell Viability Assessment

For the determination of cell viability, the bEnd.3 were seeded on Transwell^®^ inserts (pore size of 0.45μm, insert diameter of 12 mm, and an effective filtration area of 0.6 cm^2^) (Merck–Millipore, PIHA01250, 6 Hatters Ln, Watford, UK) at a density of 5 × 10^5^ cells/insert; the inserts were placed in 24-well plates (Bio-Smart Scientific, No. 30024, Park Edge Mews, Link Way, Edgemead, Cape Town, South Africa). Growth media (completed DMEM) were added to both the luminal (300 µL) and basolateral sides (800 µL), cells were incubated at 37 °C under 5% CO_2_ overnight, allowing them to attach to the surface of the filter membrane. The U-87 cells were separately seeded in 12-well plates (Bio-Smart Scientific, No. 30012) at a density of 2 × 10^5^ cells/well on the same day.

Following 24 h of incubation, bEnd.3 cells were cocultured with U-87cells by placing the inserts with bEnd.3 cells in the 12-well plates containing U-87 cells (NB: 12-well plates were used, as these wells provided a greater surface area for the U-87 cells, and thus potentiated the cancer paracrine effect). Plates were incubated at 37 °C with 5% CO_2_ for 96 h. Then, the inserts were removed from the 12-well plates and placed in new 24-well plates. This was important to ensure that only the BECs were assayed for viability and were not cross-contaminated with U-87 cells. In brief, 100 µL of XTT solution was added to each insert, and the cells were incubated for an additional 4 h at 37 °C and 5% CO_2_. Then, the media from the inserts were transferred into 96-well plates (SPL Life Sciences, No. 30096, 26, Geumgang-ro 2047 beon-gil, Naechon-myeon, Pocheon-si, Gyeonggi-do, Korea). The absorbance was measured at 450 nm by using a microplate reader (POLARstar Omega B.M.G. Labtech, Allmendgrün 8, Ortenberg, Germany).

In addition, the bEnd.3 cells were seeded in 96-well plates (SPL Life Sciences, No. 30096) at a density of 4 × 10^3^ cells/well, the cells were incubated for 24 h, and treated with U-87CM, as previously described. Following the treatment, the viability of bEnd.3 cells were determined using an XTT assay kit (Roche, No. 11465015001) in 24 h intervals up to 96 h. At each 24 h interval, 50 µL of XTT solution was added to each well, the cells were then incubated for 4 h at 37 °C in a 5% CO_2_ incubator. The absorbance was then measured at 450 nm using a microplate reader (POLARstar Omega B.M.G. Labtech).

#### 2.4.2. Mitochondrial Activity Assays

##### Mitochondrial Membrane Potential (∆ψm)

Changes in ∆ψm in bEnd.3 cells after exposure to U-87CM were analysed using tetramethylrhodamine ethylesterperchlorate (TMRE) assay (Thermo Fisher Scientific, No. T669, 168 Third Avenue, Waltham, MA, USA). TMRE is a permeable cationic, lipophilic dye, emitting red-orange fluorescent. It is taken up by active mitochondria into the negatively charged mitochondrial matrix. The intensity of the fluorescent signal obtained is indicative of the ∆ψm. The higher membrane potential (more polarised) indicates more TMRE accumulation in the mitochondrial matrix [28]. Therefore, the higher red-orange fluorescence would indicate a higher membrane potential (also called hyperpolarisation). In this assay, the bEnd.3 cells were seeded in flasks at a density of 3 × 10^4^ cells per flask and treated as previously described. In addition, bEnd.3 were also treated with carbonyl cyanide-3-chlorophenyl hydrazone (CCCP) (Sigma, Eschenstr., Taufkirchen, Germany) as a negative control. The CCCP is a classic oxidative phosphorylation uncoupler, causing the predictable decrease of the ∆ψm and was, therefore, used as a negative control. Furthermore, CCCP was used to confirm that the uptake of the TMRE was related to the mitochondrial membrane potential. At 24 h intervals, 100 µM of CCCP was added and incubated for 10 min before staining with TMRE (at 300 nM for 20 min). The solution stain was then removed, and cells were washed twice with PBS. Cells were then scraped and lysed in a lysis buffer composed of SDS (0.1% v/v) in 0.1 M Tris-HCl buffer. The 150 µL of the lysates were loaded in 96-well plates. The fluorescence of TMRE was measured with a multiwell fluorescence plate reader (POLARstar Omega B.M.G. Labtech), with excitation and emission set at 508 ± 20 nm and 589 ± 40 nm, respectively. A total protein concentration was then determined in the remaining lysate samples, corresponding in their lysates using a bicinchoninic acid (BCA) kit (Thermo Fisher Scientific, No. 232225). The fluorescence in each well was normalized for the protein concentration of its corresponding lysate.

##### ATP Generation

Relative intracellular ATP levels were determined using the Mitochondrial ToxGlo™ kit (Promega (G8000), 2800 Woods Hollow Road, Madison, WI, USA). The Mitochondrial ToxGlo™ assay was conducted according to the supplier’s protocol. An ATP detection solution was prepared by mixing 10 mL of ATP buffer with an ATP detection substrate. The components were homogenized by vortex to form an ATP detection solution.

To measure the ATP level in bEnd.3 cells cocultured with glioblastoma U-87 cells, bEnd.3 were seeded on Transwell^®^ inserts (pore size of 0.45 μm, filtration diameter of 12 mm, and an effective filtration area of 0.6 cm^2^) at a density of 2 × 10^3^ cells/insert; the inserts were placed in 24-well plates. Growth media (complete DMEM) were added to both the luminal (300 µL) and basolateral sides (800 µL), and cells were incubated at 37 °C under 5% CO_2_ overnight. The U-87 cells were separately seeded at a density of 1 × 10^3^ cells/well on the same day in 12-well plates. Following 24 h incubation, bEnd.3 on the inserts were placed in the 12-well plates where U-87 cells were growing on the well bottoms. The coculture cells were incubated at 37 °C under 5% CO_2_ for 96 h. The inserts were then removed from the 12-well plates and placed in new 24-well plates. Then, 100 µL of ATP detection solution was added to each insert. Cells were then incubated for an additional 5 min at room temperature in a plate shaker. The mixture from the inserts was transferred into white 96-well plates (SPL Life Sciences, No. 31396). The luminescence was measured using a microplate reader (POLARstar Omega B.M.G. Labtech).

In addition, bEnd.3 cells were seeded (1 × 10^3^ cells/well) in white 96-well plates (SPL Life Sciences, No. 31396) and exposed to the U-87CM, as previously described. At 24 h intervals, 100 µL of ATP detection solution was added to each well. Then, the plates were incubated for 5 min at room temperature on a plate shaker. ATP content was measured using a luminescent plate reader (POLARstar Omega B.M.G. Labtech).

#### 2.4.3. Transendothelial Electrical Resistance (TEER)

Brain endothelial monolayer integrity was tested by determining the transendothelial electrical resistance (TEER) using the EVOM TEER measurement system (EVOM: American Laboratory Trading Inc., 12 Colton Road, East Lyme, CT, USA). The bEnd.3 cells were grown on filter membranes (Transwell^®^ inserts with 0.45 µm pore size) at a density of 5 × 10^4^ cells/insert. The U-87 cells were seeded in 12-well plates. To evaluate the effect of U-87 cells on the integrity of bEnd.3 monolayer under normoxia (21% O_2_), both cell lines were first grown separately for 96 h; then, the inserts with bEnd.3 cells were placed in the 12-well plates containing the established U-87 cells. The cocultured cells were incubated in normoxic conditions, and TEER was measured daily, starting on day 2.

To evaluate the effect of U-87 cells on bEnd.3 cells under hypoxia, both bEnd.3 and U-87 cells were grown separately in similar conditions as above for 72 h, and the two cell lines were cocultured by placing the bEnd.3 inserts in the 12-well plates containing the U-87 cells. The cocultured cells were incubated in normoxic conditions (21% O_2_) for 24 h, and then incubated under hypoxic conditions (5% O_2_) for the rest of the experimental days. TEER was measured daily, starting from day 3.

To determine the effect of U-87CM on the bEnd.3 monolayer’s transendothelial electrical resistance, bEnd.3 cells were seeded on filter membranes (Transwell^®^ inserts with 0.45 µm pore size) at a density of 5 × 10^4^ cells/well in 12-well plates for 72 h, and incubated with hypoxic or normoxic U-87CM at concentrations of 20%, 40%, and 75%. Cells were then incubated at 37 °C and 5% CO_2_. TEER measurement started by day 3. In control groups, bEnd.3 cells were grown in inserts and placed in wells with completed DMEM. The measurement was performed by connecting the electrodes to either side of the cell monolayer and measuring the resistance. The resistance of the blank (inserts without cells) was subtracted from the resistance value of the cell monolayer (inserts with cells). The resultant value was multiplied by the surface area of the inserts to give the TEER value. The measurement was performed as described by Srinivasan et al., 2015 [29].

#### 2.4.4. Quantitative PCR (qPCR) Gene Expression Assay

To determine whether the reduction in the endothelial resistance of bEnd.3 cells exposed to U-87CM is associated with tight-junction proteins, qPCR was performed to quantify the gene expression of tight-junction proteins (Occludin and Claudin-5). The bEnd.3 cells were grown in 75 cm^2^ flasks and exposed daily to normoxic or hypoxic U-87CM, as previously mentioned. Following the treatment of bEnd.3 cells, a total RNA was extracted using TriPure isolation reagent (Roche, Ref:11667157001, Ground Floor Liesbeeck House River Park, River Lane, Mowbray, Cape Town, South Africa). The first strand of cDNA was synthesized from the total RNA using a Transcriptor first-strand cDNA synthesis kit (Roche, No. 04379012001). The resultant cDNA served as a template for real-time PCR amplification using a SYBR Luna Universal qPCR Master Mix kit (New England bio labs) using the real-time PCR system (Applied Biosystems real-time PCR instrument (Thermo Fisher Scientific, REF 4484643)). To amplify a fragment of Claudin-5, Occludin, and GAPDH (as the housekeeping gene), the primer pairs detailed in Table 1 were used. The amplification was conducted at 95 °C for 1 s, followed by 44 cycles of 95 °C for 15 s, 63 °C for 30 sec, and 95 °C for 15 s. Results were analysed using the Pfaffl method, as described by Pfaffl et al., 2002 [30].

### 2.5. Statistical Analysis

Statistical analysis was performed using GraphPad Prism software (version 6, GraphPad Software, San Diego, CA, USA). Data were expressed as mean ± SEM, and the differences between groups were analysed by unpaired Students’ *t*-test or one-way ANOVA, followed by Dunnett’s multiple comparison test. The significance level was accepted at *p* < 0.05 for a 95% confidence interval.

## 3. Results

### 3.1. The Effect of U-87 Cells or Their Conditioned Media (U-87CM) on the Viability of bEnd.3 Cells

**Figure 2:**
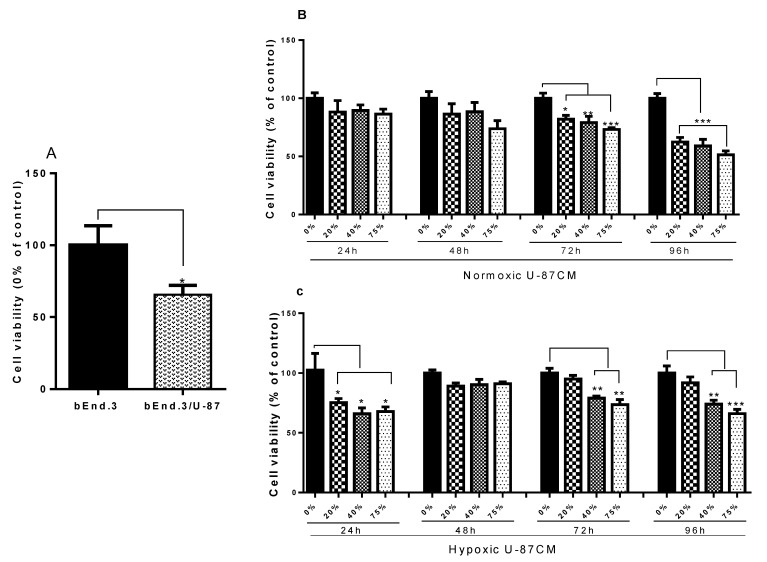
Graphs (**A**–**C**) show the viability of bEnd.3 cells under the influence of glioblastoma U-87 cells. (**A**) Shows the viability of monocultured bEnd.3 cells, compared with the bEnd.3 cells cocultured with glioblastoma U-87 cells. Cocultured cells showed a significant reduction in viability (* *p* < 0.04). (**B**) Shows cell viability of bEnd.3 cells exposed daily to selected concentrations of conditioned media produced from glioblastoma U-87 cells (U-87CM) cultivated under normoxic incubation (21% O_2_) (* *p* < 0.05, ** *p* = 0.0011, *** *p* < 0.001). The results show that the viability of cells significantly decreased at all concentrations of conditioned media at 72 and 96 h of incubation. (**C**) Represents cell viability of bEnd.3 cells after daily exposure to U-87CM derived during hypoxic (5% O_2_) incubation. Viability was significantly reduced at all concentrations of conditioned media after 24 h incubation, and only at 40% and 75% concentrations of conditioned media after 72 and 96 h incubation (* *p* < 0.05, ** *p* < 0.01, *** *p* = 0.0008), but showed no significant difference to controls across all concentrations of conditioned media at 48 h of incubation, (*n* = 4).

In the first set of experiments, the effect of coculturing glioblastoma U-87 cells on the viability of bEnd.3 cells were investigated (Figure 2A). Using an XTT cell viability assay, bEnd.3 cell viability was significantly reduced after coculturing with U-87 cells (*p* < 0.04) under normoxic conditions. Following 72 and 96 h of exposure to selected concentrations of U-87CM (produced from U-87 cells cultivated under normoxic or hypoxic conditions), mostly prolonged exposure (72 and 96 h) of both hypoxic and normoxic U-87CM reduced cell viability of bEnd.3 cells, compared to the control (Figure 2B,C). At 24 h of exposure to hypoxic U-87CM, a significant reduction was observed in bEnd.3 cell viability (*p* < 0.05) (Figure 2C). However, treatment with normoxic U-87CM at 24 h produced a slight non-statistical suppression of bEnd.3 cell viability (Figure 2B). At 48 h of exposure to both normoxic- (Figure 2B) and hypoxic- (Figure 2C) derived U-87CM, no statistically significant difference was observed in the viability of bEnd.3 cells. The effects at 96 h were more prominent under normoxic conditions, compared to hypoxic conditions, and the reduction of cell viability depended on both the length of the exposure time to the treatment and the concentration of U-87CM.

### 3.2. The Effect of U-87CM on the Mitochondrial Activity of bEnd.3 Cells

The viability experiments showed that bEnd.3 cells were affected by the U-87CM paracrine secretome. In light of these viability data, additional experiments were performed to obtain more detailed data on the mechanism of the observed effect of U-87CM on the mitochondrial activity of bEnd.3 endothelial cells. The ability of U-87CM to affect the mitochondrial activity in bEnd.3 cells were investigated through the evaluation of mitochondrial membrane potential and ATP levels.

#### 3.2.1. Mitochondrial Membrane Potential (ΔΨm)

**Figure 3:**
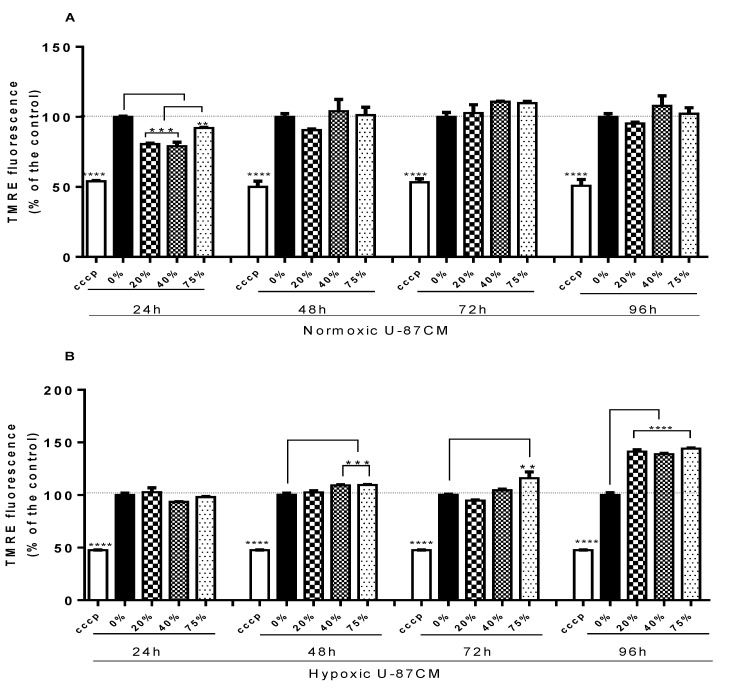
Graphs (**A**,**B**) show the changes in mitochondrial membrane potential (ΔΨm) in bEnd.3 cells under the influence of glioblastoma U-87 cell-derived CM. (**A**) Shows mitochondrial membrane potential (ΔΨm) in bEnd.3 cells after daily exposure to selected concentrations of glioblastoma U-87 conditioned media (U-87CM) derived from U-87 cells under normoxic conditions (21% O_2_). (**B**) Shows the changes in mitochondrial membrane potential (ΔΨm) in bEnd.3 cells after daily exposure to selected concentrations of conditioned media derived from U-87 cells under hypoxic conditions (5% O_2_). CCCP: carbonyl cyanide 3-chlorophenylhydrazone, a known Ψm depolarising agent, was used to decrease the mitochondrial membrane potential (negative control) (** *p* < 0.01, *** *p* < 0.001, **** *p* < 0.0001), (*n* = 4).

The changes in the mitochondrial membrane potential were measured by using a tetramethylrhodamine ethylesterperchlorate (TMRE) assay. The stain of bEnd.3 cells with TMRE was performed in parallel with carbonylcyanide-3-chlorophenylhydrazone (CCCP). CCCP was used to produce a decrease in the ΔΨm through oxidative phosphorylation uncoupling, and formed the negative control. The exposure of bEnd.3 cells to normoxic U-87CM produced a statistical decrease in the ΔΨm activity in all treatment concentrations at 24 h of exposure (Figure 3A) (** *p* < 0.01, *** *p* < 0.001). The decrease in ΔΨm indicated depolarization of ΔΨm, although the depolarization state was not constant, as cells recovered statistically to control ΔΨm at 48, 72, and 96 h exposure. However, bEnd.3 cells exposed to hypoxic U-87CM showed a significant increase in the ΔΨm activity after 48 h of treatment, particularly after 48 h exposure (at 40% and 75%; *p* < 0.001), at 72 h (at 75%; *p* < 0.01), and at 96 h, at all treatment concentrations (*p* < 0.0001), compared to the control (Figure 3B).

#### 3.2.2. ATP Level in bEnd.3 Cells under the Influence of Glioblastoma U-87 Cells

**Figure 4:**
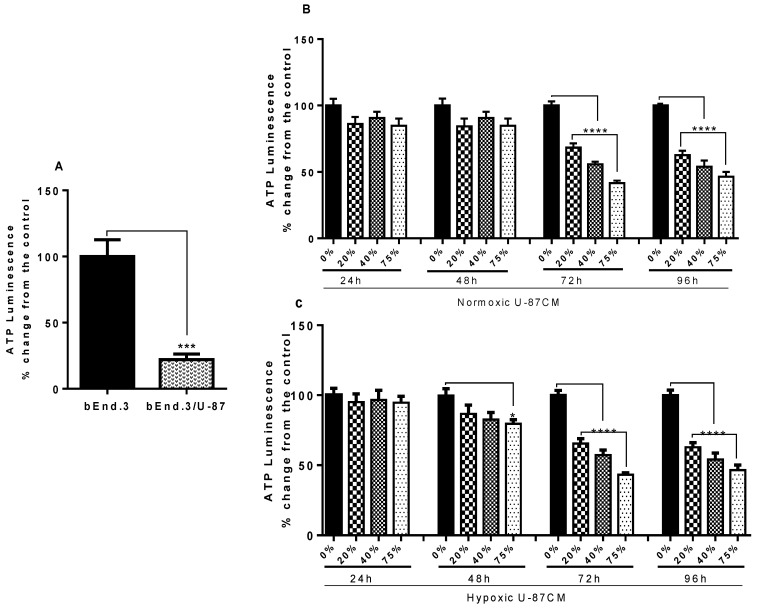
Graphs (**A**–**C**) show the changes in cellular ATP levels in bEnd.3 cells under the influence of glioblastoma U-87 cells. (**A**) Shows the significant reduction in cellular ATP level in bEnd.3 cells cocultured with glioblastoma U-87 cells, compared to monocultured bEnd.3 cells (*** *p* = 0.0004), (*n* = 4). (**B**) Shows significant reduction in cellular ATP levels in a dose–response manner, compared to the control, after 72 and 96 h in culture in normoxic U-87CM (derived from U-87 cells cultivated in normoxic conditions (21% O_2_) (**** *p* < 0.0001)), (*n* = 4). (**C**) Shows significant reduction in cellular ATP levels in a dose–response manner, compared to the control, after 72 and 96 h after treatment with selected concentrations of hypoxic U-87CM (derived from U-87 cells cultivated in hypoxic conditions (5% O_2_) (* *p* = 0.0385; **** *p* < 0.0001), (*n* = 4).

The primary function of mitochondria is to generate ATP through oxidative phosphorylation. ATP levels in bEnd.3 cells cocultured with glioblastoma U-87 cells or their conditioned media (U-87CM) were investigated. Results showed a significant decrease in ATP levels in bEnd.3 cells after being cocultured with U-87 cells (Figure 4A) (*p* < 0.001). Similar results were observed after long-term exposure (for 72 and 96 h) of bEnd.3 cells to both normoxic and hypoxic U-87CM (Figure 4B,C; *p* < 0.0001); the cellular ATP levels were decreased in a dose-dependent manner. However, no significant difference was observed after 24 or 48 h treatment with either normoxic or hypoxic U-87CM (Figure 4B,C), except at 48 h exposure to 75% hypoxically derived U-87CM, which showed a significant decrease in the ATP level, relative to the control (*p* = 0.0385).

### 3.3. The Effect of the Coculture of Glioblastoma U-87 Cells on the Permeability of bEnd.3 Cell Monolayer

The ability of U-87 cells or their conditioned media (U-87CM) to perturb the permeability of the bEnd.3 cells monolayer was assessed by measuring the transendothelial electrical resistance (TEER) across confluent monolayers of bEnd.3 cells. The bEnd.3 cells were cocultured with U-87 cells under hypoxic and normoxic conditions using the Transwell system, or cultivated with hypoxic- and normoxic-derived U-87CM at selected concentrations (20%, 40%, and 75%), as previously described.

**Figure 5:**
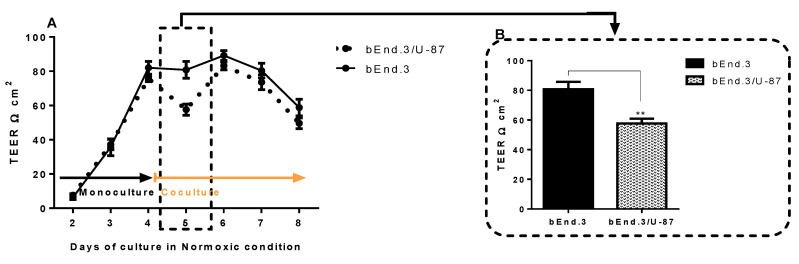
Transendothelial electrical resistance (TEER) of bEnd.3 monolayers. (**A**) Confluent monocultures of bEnd.3 cells were cocultured with glioblastoma U-87 cells under normoxic conditions (21% O_2_). TEER was only statistically different to control monolayers on day 5, and on all other days were statistically not different (*p* > 0.05). (**B**) Peak-TEER of bEnd.3 monolayers after 24 h coculture with glioblastoma U-87 cells (** *p* = 0.002), (*n* = 3).

TEER across bEnd.3 confluent monolayers cocultured with U-87 cells under normoxic conditions is shown in Figure 5A. TEER across monocultured bEnd.3 monolayers (controls) and the cocultured bEnd.3 monolayer (bEnd.3 cells cocultured with glioblastoma U-87 cells) were measured on a daily basis for 8 days.

It is important to note that, from days 2–4, both control and experimental groups of bEnd.3 cells were grown as monocultures. Furthermore, TEER readings typically increased with time, and no difference was observed between the two groups of cells. By the end of day 4, cells in the control group were kept as a monoculture. In contrast, the experimental bEnd.3 monolayers were cocultured with U-87 cells. The bEnd.3 monolayers cocultured with U-87 cells had significantly reduced TEER measurements after 24 h, as compared to the controls (Figure 5A,B on day 5) (*p* = 0.002). Interestingly, the reduction of TEER was not constant, as bEnd.3 cells cocultured with U-87 cells recovered their resistance by day 6 and were statistically not different from controls at days 6 to 8.

**Figure 6:**
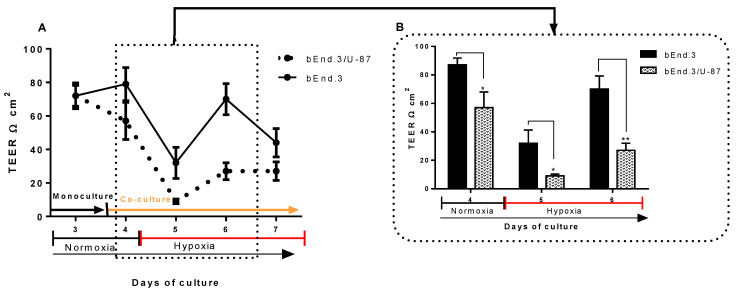
Transendothelial electrical resistance (TEER) of bEnd.3 monolayers. (**A**) Transendothelial electrical resistance (TEER) of bEnd.3 cells under hypoxic coculture with U-87 cells. Note that monocultures of bEnd.3 cells were introduced to coculture with U-87 cells after day 3 under normoxic conditions (TEER reading on day 4); thereafter, hypoxic conditions were introduced (after day 4 to the end of the experimental timeframe). (**B**) Illustrates the changes of TEER of bEnd.3 cells from normoxic to hypoxic incubation (* *p* < 0.05, ** *p* < 0.002), (*n* = 3).

To compare the effects of coculture of U-87 cells under hypoxic conditions, bEnd.3 monolayers were cultured under normoxic conditions and as monocultures until day 3 (Figure 6). TEER measurements were carried out on these monocultured bEnd.3 cell monolayers incubated under normoxic conditions until day 3, whereafter, experimental bEnd.3 monolayers were cocultured with U-87 cells under normoxic conditions (21% O_2_) for 24 h (Figure 6, day 4). Hypoxic incubation started by the end of day 4, and TEER was measured under these conditions for the rest of the experimental timeframe (Figure 6, 7 days).

As shown in Figure 6A, under normoxia, the coculture with U-87 cells significantly decreased compared to the control (Figure 6A,B on day 4) (*p* = 0.032). The reduction in the resistance of the cocultured bEnd.3 monolayer was constantly suppressed on the days after hypoxic incubation (Figure 6A,B on day 5) (*p* = 0.033). Interestingly, the resistance of the bEnd.3 monoculture (control) also decreased in hypoxia, although it recovered by day 6 to normal levels of TEER (Figure 6B, day 6) (*p* < 0.002). Although the TEER of the controls (bEnd.3 cell monolayers) and the cocultured bEnd.3/U-87 cells were suppressed under conditions of hypoxic coculture, both recovered on day 6, but the recovery of the monocultured bEnd.3 cell monolayers were much greater.

### 3.4. The Effect of the U-87CM on the Permeability of the bEnd.3 Cell Monolayer

**Figure 7:**
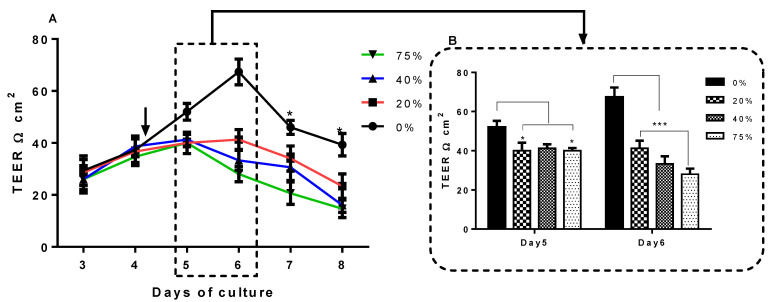
TEER of bEnd.3 cells exposed to selected concentrations of U-87CM. (**A**) TEER of bEnd.3 monolayer exposed to U-87CM produced from U-87 cells in normoxic conditions (21% O_2_). The arrow indicates the start of monolayer treatment with U-87CM at the end of day 4. The asterisks (*) refer to the significant difference between controls (0%) and cells exposed to selected concentration of normoxic U-87CM; on day 7 (at 40% and 75%; * *p* < 0.05, *** *p* < 0.001, respectively); on day 8 (at 20% (* *p*< 0.05), at 40%, and 75% (*** *p*< 0.001)). (**B**) Compares “peak” TEER values across bEnd.3 monolayers on days 5 and 6 after exposure (for 24 and 48 h, respectively) to U-87CM from the normoxic conditions (* *p* < 0.05, *** *p*< 0.001), (*n* = 3).

The exposure of bEnd.3 cells to U-87CM negatively affected the permeability of the bEnd.3 monolayers. The bEnd.3 confluent monolayers were exposed to selected concentrations of normoxically derived U-87CM (Figure 7A). The treatment with normoxic U-87CM started on day 4 of cultivation. On day 5, barrier resistance of the bEnd.3 cell monolayers decreased after 24 h of exposure, particularly at 20% and 75% concentrations (*p* < 0.05). However, the exposure of the bEnd.3 monolayer to the 40% concentration showed a nonstatistical decrease in TEER, in comparison with the control (0%). On day 6, exposed bEnd.3 cell monolayers had significantly decreased TEER measurements at all treatment levels, relative to the control (Figure 7B, day 6) (*p* < 0.001). This statistically significant trend continued on days 7 and 8.

**Figure 8:**
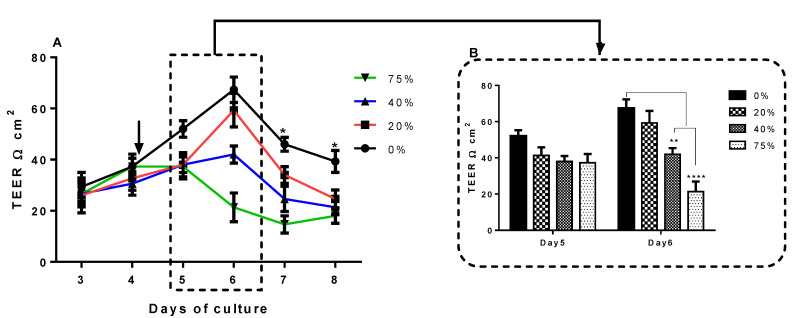
TEER of bEnd.3 cells exposed to selected concentrations of U-87CM. (**A**) Shows TEER of bEnd.3 monolayers exposed to hypoxic U-87CM. The arrow indicates the start of treatment of monolayers with U-87CM at the end of day 4. The asterisks (*) refer to the significant difference between controls (0%) and cells exposed to selected concentrations of hypoxic U-87CM; on day 7 (at 40% and 75%; ** *p* < 0.01, **** *p* < 0.0001, respectively) and on day 8 (at 20%, 40%, and 75%; * *p* < 0.05, ** *p*< 0.01, *** *p* < 0.001, respectively). (**B**) Compares “peak” TEER on day 5 (24 h exposure) and day 6 (48 h exposure) (** *p* < 0.01, **** *p*< 0.0001), (*n* = 3).

Using the same experimental design as in the normoxia permeability experiments, hypoxic U-87CM also decreased the barrier resistance (Figure 8A). The difference in TEER across the control (0%) and the bEnd.3 monolayers exposed to U-87CM concentrations at day 5 (after 24 h exposure) was a nonstatistical decrease across the treatment concentration range (Figure 8B, day 5). However, 24 h later, on day 6, hypoxic U-87CM had a significant decrease in the barrier resistance in a concentration-dependent manner (Figure 8B) at 40% (*p* < 0.01) and 75% (*p* < 0.0001), respectively. However, the resistance of bEnd.3 monolayers exposed to 20% U-87CM at day 6 was not significant, in comparison to the control.

### 3.5. qPCR Gene-Expression Analysis

**Figure 9:**
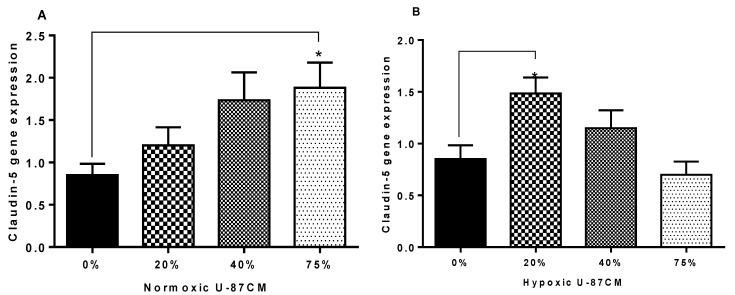
Gene expression level for Claudin-5 in bEnd.3 cells under the paracrine influence of glioblastoma U-87 cells. (**A**) Shows Claudin-5 gene expression in bEnd.3 cells after the exposure to selected concentrations of conditioned media derived from U-87 cells (U-87CM) under normoxic conditions (21% O_2_) (* *p* < 0.03). (**B**) Represents Claudin-5 gene expression in bEnd.3 cells after the exposure to selected concentrations of U-87CM were generated under hypoxic conditions (5% O_2_) (* *p* < 0.05), (*n* = 4).

To clarify the mechanism by which glioblastoma U-87CM perturbs the resistance across the bEnd.3 monolayer, gene expression of tight junctions (Occludin and Claudin-5) in bEnd.3 cells exposed daily to U-87CM was evaluated using qPCR. As demonstrated in Figure 9A, the exposure to normoxic U-87CM increased Claudin-5 gene expression in bEnd.3 cells. However, only the high concentration (75%) of normoxic U-87CM significantly elevated the gene expression of Claudin-5 (*p* < 0.03). The expression of the Claudin-5 gene was significantly increased in bEnd.3 cells exposed to hypoxic U-87CM (Figure 9B). However, bEnd.3 cells exposed to a low concentration of hypoxic U-87CM (20%) showed the highest gene expression of Claudin-5, relative to the control (*p* < 0.05), whereas the exposure to the other concentrations (40% and 75%) did not show a significant difference in Claudin-5 gene expression, compared to the control.

**Figure 10:**
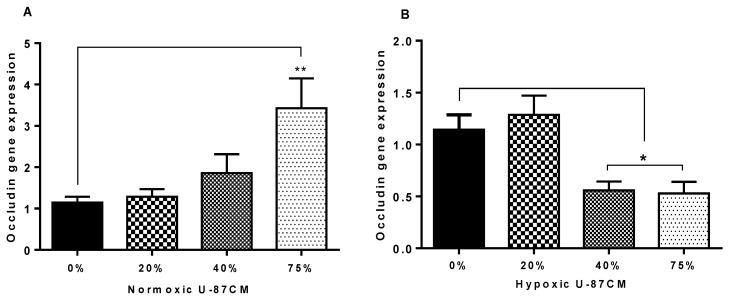
Gene expression level for Occludin in bEnd.3 cells after the exposure to selected concentrations of U-87CM. (**A**) Shows Occludin gene expression in bEnd.3 cells that were exposed daily to U-87CM produced in normoxic conditions (21% O_2_) (** *p* = 0.0047). (**B**) Represents gene expression of Occludin in bEnd.3 exposed daily to U-87CM produced under hypoxic conditions (5%O_2_) (* *p* < 0.05), (*n* = 4).

Occludin gene expression was also quantified in bEnd.3 cells cultivated in U-87CM. The bEnd.3 cells treated with the 75% concentration of normoxic U-87CM had significantly increased levels of Occludin gene expression, as compared to the controls (Figure 10A) (*p* < 0.0047). However, no significant difference was observed in Occludin gene expression in bEnd.3 cells treated with 20% or 40% concentrations of U-87CM, in comparison to the control. The bEnd.3 cells exposed to 40% and 75% concentrations of hypoxic U-87CM showed a significant decrease in Occludin gene expression (Figure 10B) (*p* < 0.05); however, the lowest treatment concentration (20%) of hypoxic U-87CM did not elicit a change in Occludin gene expression in bEnd.3 cells.

## 4. Discussion

The brain endothelial cells (BECs) are the anatomical sites governing the transepithelial transport function of the BBB [31]. The regulatory effectiveness of BECs is frustrated by brain tumours, particularly by high-grade brain tumours such as Glioblastoma [24]. Glioblastoma, also called Glioblastoma multiforme (GBM), is the most malignant form of a primary brain tumour [8], characterized by an extraordinary ability to infiltrate the surrounding neural tissue [9]. The GBM microenvironment is composed of other brain cells, including infiltrative immune cells, astrocytes, pericytes, and endothelial cells [21]. The interaction between GBM cells and other cells in the GBM environment occurs via soluble paracrine factors secreted into the GBM environment [32]. Analysis of GBM secretion showed that GBM can secret approximately 2000 variant proteins into their environment [33]. GBM-secreted factors could transiently alter both normal neural precursor cells [34] and modulate brain endothelial properties [25]. GBM-secreted factors diffuse to the surrounding cells and affect their normal physiological functionality. Constant exposure of brain endothelial cells to the tumour environment induce both phenotypic and functional alterations in BECs [23]. It is well established that, clinically, the most disruptive feature of GBM is the increased BBB permeability, which leads to the formation of oedema and the increase of the intracerebral pressure due to the disruption of the BBB, primarily at the level of the endothelial cell site [3,13].

In the current study, the effect of glioblastoma-secreted paracrine factors on the physiological state of brain endothelial cells (bEnd.3) was investigated. The study aimed to determine whether endothelial cells exposed to glioblastoma U-87 cells are physiologically altered by focusing on the mitochondrial function of BECs. Using the XTT assay, the cell viability of bEnd.3 cells cocultured with glioblastoma U-87 cells or cultivated with conditioned media produced under normoxic (21% O_2_) or hypoxic (5% O_2_) conditions was determined. The XTT cell viability assay reflects the metabolic status of a cell culture population by monitoring the changes in the activity of the mitochondrial dehydrogenase. Current results illustrated that the viability of brain endothelial cells is negatively affected by U-87 cells (Figure 2A) and by conditioned media derived from U-87 cells (Figure 2B,C). Contrary to these results, it has been previously reported that glioblastoma cells release various factors, such as growth factors [20,33], which are known to enhance the survival and angiogenic properties of BECs [35]. In addition, the cytokines that are largely expressed by GBM [36], IL-8, and Il-6 are upregulated [37]. In support of our findings of GBM-induced suppression of BEC viability, these cytokines were found to disrupt the brain endothelial function, and to induce apoptosis [38] or necroptosis [39] in endothelial cells to facilitate the extravasation of cancer cells. Previous studies focused on the endothelial alteration in the brain tumour environment (in vivo or in vitro) by establishing models that monitor the interaction between cancer cells and endothelial cells, mostly focusing on the molecular and morphological aspects. However, none have focused on the metabolic alteration of BECs under the influence of GBM. In the current study, the endothelial metabolic activity was significantly reduced following daily exposure to glioblastoma U-87 cells and their secretions, as indicated by the reduction in the viability of bEnd.3 cells cultivated with glioblastoma U-87 cells or their conditioned media (U-87CM). As the bEnd.3′s suppressed viability was monitored by measuring mitochondrial dehydrogenase activity, it implicated the modulation of BEC mitochondrial function via GBM paracrine factors.

The intact BBB is as rigorous in preventing the efflux of unsolicited cells/substances as it is in preventing their influx into the brain [40]. It is, therefore, in the interest of the metastatic tumour to compromise the integrity of the BBB to relocate to non-neural tissue. The present study showed that these normoxic paracrine factors were not effective within 48 h but only affected BEC viability at 72 h, while having a more pronounced dose effect at 96 h. This alludes to these paracrine factors having an insidious long-term effect on the mitochondrial function, which only becomes statistically evident from 72 h onward.

It appears that under hypoxic conditions that the paracrine effect is more aggressive within 24 h of exposure, and despite a nonstatistical decrease at 48 h, the suppressive trend was clearly present at 72 and 96 h. However, the effects of U-87CM were unexpectedly more pronounced under normoxic conditions, especially in the long-term (at 96 h) treatment. This may allude to the more aggressive GBM secretion of paracrine factors under normoxic conditions, relative to hypoxic conditions. The clear dose-related effect of U-87CM in both normoxic and hypoxic treatments further indicates that blocking this paracrine effect may be a possible avenue for clinical intervention.

To further investigate the mitochondrial activity in bEnd.3 cells under the influence of U-87 glioblastoma cells, we evaluated the mitochondrial membrane potential (ΔΨm) of bEnd.3 cells after daily exposure to normoxic and hypoxic U-87CM, by staining bEnd.3 cells with tetramethylrhodamine ethylesterperchlorate (TMRE). Physiologically, mitochondria use the electrochemical driving force of protons (H^+^) produced from the reductive transfer of electrons through protein complexes I–IV in the inner mitochondrial membrane to produce ATP [41]. The differential concentration of protons between the outer mitochondrial space and the mitochondrial matrix forms the basis of the mitochondrial membrane potential (ΔΨm). This process accumulates H^+^ in the outer intermitochondrial space, which subsequently flows back into the mitochondrial matrix via the ATP-producing F1/F0 ATP-synthase to complete the electron transport chain and to, thereby, generate ATP. A low ΔΨm indicates a decreased driving force for ATP and vice versa [28,42].

The bEnd.3 cells exposed to normoxically derived U-87CM showed suppression in the ΔΨm, compared to the control, only at 24 h. Thereafter, at 48–96 h, no statistically significant difference was observed, compared to the controls (Figure 3A). In contrast, an increase in the ΔΨm (hyperpolarisation) was observed in bEnd.3 treated with hypoxic U-87CM after long-term exposure, particularly 96 h, where all treatments with hypoxically derived U-87CM produced significantly higher ΔΨm (Figure 3B). At the cellular level, this indicated an accumulation of TMRE (H^+^) in the intermitochondrial space, yielding higher fluorescent intensity than in control bEnd.3 cells. The hyperpolarisation state results from the high transfer of H^+^ to the outer intermitochondrial inner space or from a compromise of the H^+^ driving force via the ATP-synthase. ATP-synthase inhibition reduces the utilization of the electrochemical H^+^ gradient, which causes ATP depletion, ADP accumulation, and ΔΨm hyperpolarisation [43]. Although the current data implicate hypoxically derived U-87CM in modulating the state of ΔΨm, further investigation is recommended to determine the exact mechanism for mitochondrial hyperpolarisation in brain endothelial cells in a GBM cancer environment.

Given the effects of normoxic and hypoxically derived U-87CM on ΔΨm, the current study investigated how ATP levels in the bEnd.3 BECs would be affected. Therefore, cellular ATP levels in bEnd.3 cells exposed to glioblastoma U-87 cells (coculture) or their conditioned media (U-87CM) were measured as a further evaluation for mitochondrial function. Data derived from the current study showed a marked reduction of ATP levels in bEnd.3 cells after a daily coculture with glioblastoma U-87 cells (Figure 4A). Similar results were observed after long-term exposure to normoxic and hypoxic U-87CM (Figure 4B,C, respectively), where suppression of ATP levels was nonstatistical at 48 h, but at 72 and 96 h, a clear dose-related suppression of ATP concentration in the BECs was observed. This was in alignment with the decrease in bEnd.3 cell-viability observations (Figure 2), and the elevated ΔΨm by hypoxically derived U-87CM seen at 96 h (Figure 3B). Given that the main function of mitochondria is ATP synthesis, and that most physiological activities of the cell are dependent on the availability of ATP, it is an accurate indicator of mitochondrial activity and cell viability [44]. In the current study, the depletion in ATP levels under hypoxic conditions may be associated with a GBM-induced increase in ΔΨm in BECs, uncoupling the process whereby the H^+^ concentration in the inter-mitochondrial space drives the production of ATP via ATP-synthase [45]. In contrast, the normoxically induced reduction in ATP levels was not related to hyperpolarisation of ΔΨm, which suggests that under normoxia, GBM induces decreased ATP levels via another mechanism. Given that ATP levels in the cell are related to the reversible reactions of ATP synthesis and hydrolysis, ATP + H2O <=> ADP + (Pi), we postulate that, in view of the fact that no hyperpolarization/depolarization of the outer-mitochondrial space (ΔΨm) was observed during normoxic conditions (Figure 3A), the hypothetical proton-driven suppression of ATP concentrations at 72 h and 96 h is not valid. It is, therefore, indeed plausible that the mechanism of ATP depletion involved increased hydrolysis of ATP, rather than suppressed proton-driven ATP synthesis. Given the limitations of this study, it is recommended that further study be carried out to elucidate the effect of cancer paracrine factors under normoxic and hypoxic conditions on ATP synthesis and hydrolysis in conjunction with O_2_ consumption.

One of the most disruptive features of clinical GBM is increased BBB permeability and brain oedema formation. The disruption in cellular ATP level has also been associated with BBB permeability and tight-junction protein changes in mice [46]. Thus, the current study investigated the effects of coculturing bEnd.3 monolayers with U-87 GBM cells, as well as treating bEnd.3 monolayers with both normoxically and hypoxically derived U-87CM on the transendothelial permeability (using TEER). The coculture experiments conducted under normoxic conditions in the current study demonstrated a statistically transient increase in permeability (decrease TEER) (at 24 h) across bEnd.3 monolayers (Figure 5). However, after 24 h, the permeability returned to TEER levels that were not statistically different from controls. These data are in alignment with our data on normoxic ΔΨm, where we also only saw a significant depression at 24 h but not thereafter. This transient decrease in permeability under normoxic conditions may not be sufficient for GBM tumour cells to metastatically escape from neural tissue across the notoriously impermeable BBB, and it may be the reason for the low level of non-neural metastasis observed in patients with GBM tumours (<2%) [47]. However, clinically the progression of GBM is often associated with the migration of GBM tumour cells along nerve tracts and along the outer perimeters of blood vessels within the CNS [48]. Nevertheless, there is clear evidence in the literature that GBM tumours located in the CNS can metastatically cross the BBB (most likely in cases where the BBB has been compromised) and relocate to bone tissue, lung tissue, and muscle tissue [49]; however, this only reflects 2% of all GBM cases. Furthermore, given the demand for supplying nutrients and O_2_ to neural tissues, and the high density of blood vessels in brain tissue, where the extravascular space represents only 20% of the neural tissue [48], it is unlikely that the GBM tumour’s microenvironment would be in a state of hypoxia. Thus, it may only be under conditions of the rapid growth of the GBM tumour that intratumour tissue may be hypoxically challenged. It is under these hypoxic conditions that differential modulation of BECs may occur.

In the experiment presented in Figure 6, two sets of bEnd.3 monolayers were cultured for 48 h (2 days) under normoxic conditions, and on day 3, TEER was measured. Immediately afterwards, one set of the bEnd.3 monolayers (on inserts) was introduced to coculture with U-87 cells (here cancer cells were grown on the floor of the well, while the insert with the bEnd.3 monolayers was placed into this well). TEER was measured on day 4, and this allowed for the comparison of the bEnd.3 monolayer’s permeability before and after being introduced to the U-87 coculture and also to the bEnd.3 monoculture, which served as a control. Hereafter, both sets of bEnd.3 monolayers were introduced to hypoxic (5% O_2_) incubation for the rest of the experimental timeframe (until day 7), and TEER was measured on a daily basis. This allowed for the comparison of monocultured bEnd.3 monolayers with those cocultured with cancer U-87 cells, under hypoxic conditions.

In contrast to normoxic conditions seen in the coculture experiments in Figure 5, the bEnd.3 monolayers that were cocultured with U-87 cells under hypoxic conditions showed increased permeability (decreased TEER) throughout the hypoxic period (Figure 6). Thus, a clear difference in the modulation of BECs under hypoxic conditions was observed, with bEnd.3 monolayers cocultured with U-87 cells remaining highly permeable throughout the hypoxic conditions. It is unlikely that the increased permeability was related to the decreased levels of O_2_, as it must be pointed out that hypoxia only depressed monocultures of bEnd.3 cells transiently, and after 24 h, these monolayers recovered their TEER values and were always statistically more impermeable, compared to bEnd.3 monolayers cocultured under hypoxic conditions (Figure 6B).

By hypothetical extension, the bEnd.3 monolayers were treated with selected concentrations of U-87CM in the current study and TEER was measured. The exposure of monocultured bEnd.3 monolayers to normoxic U-87CM (Figure 7) significantly decreased TEER in a time-dependent manner after 24 h and 48 h of treatment.

Similarly, the treatment with hypoxic U-87CM (Figure 8) decreased TEER of bEnd.3 monolayers, but only after 48 h of exposure to 40% and 75% hypoxic U-87CM. Interestingly, the resistance of the bEnd.3 monolayer was more compromised after the treatment with normoxic U-87CM (Figure 7). That might reflect the differential metabolic state of GBM cells under normoxia and hypoxia. Such differences were observed by Emily Chen et al., 2018, who reported that U-87 cells reduced their metabolic activity and proliferation under hypoxia [50]. GBM cells, like all cancer cells, actively grow close to the blood vessels due to the high level of O_2_. In addition, cancer cells under aerobic conditions preferentially use glycolysis (Warburg effect) [51]. This suggested that GBM cancer cells were highly active under normoxia, which was reflected in their paracrine secretions. At this point, the identification of factors secreted in U-87 secretions (produced in normoxia and hypoxia) will be helpful, and more research is required to elucidate these mechanisms.

The bEnd.3 cells exposed to normoxic and hypoxic U-87CM (Figure 7 and Figure 8, respectively) were unable to recover their resistance after the treatment; however, the cocultured bEnd.3 cells under normoxia (Figure 5) recovered after 48 h to the control levels of TEER, but not under hypoxia. This may be due to the coculture effect between bEnd.3 cells and U-87 cells. The coculture with endothelial cells ensured the constant cross-talk between U-87 and bEnd.3 cells. This “cross-talk” between both types of cells might lead to the modulation of paracrine or autocrine factors secreted from both cell types. It is, therefore, important to note that U-87CM were produced from monocultured U-87 cells in the absence of intermodulation between U-87 cells and bEnd.3 cells. Therefore, modulatory “cross-talk” paracrine factors between U-87 cells and bEnd.3 cells were not present in the normoxic U-87CM TEER experiments (Figure 7), where the increase in permeability (reduction in TEER) was seen throughout the treatment of U-87CM. Based on this postulate, BECs may be able to respond to U-87 cancer cell paracrine-induced increases in permeability by inhibiting the U-87 cell paracrine effects (Figure 5). In the absence of inhibition by bEnd.3 cells on U-87 cells, continuous suppression of TEER by paracrine factors in U-87CM occurs, as can be seen in Figure 7.

In the analogous coculture experiments (Figure 6), hypoxic conditions caused an extended reduction in TEER. We suggest that either the reciprocal inhibition by bEnd.3 cells on paracrine factors secreted by U-87 cells (seen in Figure 5) was suppressed under hypoxic conditions, or under hypoxic conditions U-87, cells secrete paracrine factors more aggressively. The former hypothesis is favoured in view of the fact that this paracrine effect was suppressed when U-87 cells were incubated under hypoxic conditions for the following reasons: firstly, in contrast to the analogous normoxia experiments (Figure 7), no statistical suppression of TEER occurred on day 5 (Figure 8). Secondly, in contrast to normoxically derived U-87CM, hypoxically derived U-87CM only caused a significant decrease in TEER, relative to controls, only at higher treatment concentrations (40% and 75%) on day 6. Although a more aggressive hypoxic response from U-87 cells was expected, normoxia tended to produce more prominent or aggressive responses from GBM. This may be because GBM originates from glial CNS cells in a location that is highly vascularized (80% of extracellular CNS tissue is made up of blood vessels) [52]. Therefore, in view of the fact that GBM develops in an environment that is seldom deficient in O_2_ and nutrients, it is less likely to be aggressive under hypoxic conditions. In the event of the fast-growing GBM tumour outstripping angiogenesis and developing zones of hypoxia, a process that facilitates intra-CNS metastasis, the metastatic GBM cell will always find itself in a relatively O_2_-rich CNS environment, making aggressive hypoxic paracrine mechanisms superfluous. Furthermore, the short window provided to compromise the BBB permeability under normoxic conditions (Figure 5) supported the clinical case data in that most GBM tumours seldom metastasize to extra-CNS tissue.

Endothelial resistance is maintained by transmembrane tight junctions [53], particularly in Occludin and Claudin-5 [54,55]. These proteins are the most integral tight-junction proteins at the brain endothelial paracellular space [56]. The expression of Claudin-5 and Occludin is critical for endothelial integrity. Downregulation of these proteins would lead to decreased brain transendothelial resistance [57]. A similar effect on the bEnd.3 monolayer was observed when exposed to U-87CM in the current study (Figure 5, Figure 6, Figure 7 and Figure 8). These results are consistent with a previous study by Schneider et al. (2004) that reported the impairment of the endothelial barrier by glioblastoma cells. The disruption of BBB function was also shown in parallel in vivo and in vitro studies using different types of glioblastoma cell lines [58]. Furthermore, it has been reported that GBM cells release a wide range of vascular growth factors (VGFs) [59], which in turn modulate the expression of Claudin-5, promoting the BBB impairment [60].

As Occludin and Claudin-5 are the anchors of transendothelial cell permeability, the mechanism by which glioblastoma U-87 cells decreased the resistance of the bEnd.3 monolayer was investigated in the current study. The qPCR was used to quantify the gene expression of Occludin and Claudin-5 in bEnd.3 exposed to U-87CM. The results showed statistical upregulation in gene expression for both Claudin-5 and Occludin in bEnd.3 cells exposed to 75% normoxic U-87CM (Figure 9A and Figure 10A). The Claudin-5 gene expression was statistically upregulated in bEnd.3 cells that were exposed to hypoxically derived U-87CM only at 20% concentration (Figure 9B), whereas high concentrations significantly suppressed Occludin gene expression in bEnd.3 cells (Figure 10B), endorsing our TEER data for hypoxically treated bEnd.3 monolayers with U-87CM (40% and 75%) (Figure 8). However, these results did not fully correlate with the TEER results, which engenders the following points: firstly, tight-junction proteins may be degraded by soluble factors secreted by glioblastoma cells, particularly metalloproteinases (MMPs), as it is reported that glioblastoma cells release a wide range of these proteases, degrading the tight-junctional proteins [61]. Secondly, a negative correlation between mRNA and protein expression at the level of transcription can indicate that modulation of TJs occurs post-translationally, affecting and compromising their insertion or functionality at the membrane level [62]. Lastly, the disruption of the endothelial resistance could be via the transcellular way, rather than the paracellular way. Therefore, further investigation is required to elucidate the exact mechanism whereby endothelial tight junctions and transendothelial permeability is affected by paracrine factors.

## 5. Conclusions

GBM cells modulate the function of brain capillary endothelial cells via paracrine factors differentially under normoxic and hypoxic conditions (Table 2). Furthermore, the differential permeability (TEER) effects between coculturing bEnd.3 cells with U-87 cells, compared to treating them with U-87 conditioned media, suggest cross-modulatory effects between these cell types, which strongly advocate further research. It is clear that U-87 cells and their paracrine secretions modulate ATP generation by uncoupling ΔΨm under hypoxic conditions, but do not use the same mechanism to uncouple ATP generation under normoxic conditions. Under normoxic conditions, the authors speculate that increased ATP hydrolysis might be responsible for the suppression of ATP levels in cells exposed to selected concentrations of the U-87 GBM secretome. Nevertheless, the U-87 cell-derived conditional media-induced suppression of ATP levels in the bEnd.3 is correlated with reduced viability of BECs. This long-term mechanism (only achieving statistical resolution in viability after 72 h), together with U-87 GBM cancer-cell paracrine effects causing increased permeability (decrease TEER), may be the mechanism behind the clinical oedema formation, and the subsequent increase of the intracerebral pressure, implicating the disruption of the BBB, primarily at the level of the endothelial cells of the brain capillaries. Lastly, the dose-response effect of treating BECs with different concentrations of U-87CM suggests additional avenues of research with clinical implications for treating GBM cancer patients.

## Figures and Tables

**Table 1:** Primer sequences for quantitative PCR (qPCR) amplification of complementary DNA (cDNA). GAPDH: glyceraldehyde phosphate dehydrogenase.

N	Primers	Primer Pairs (Sequence (5′ > 3′))	Product Length	°C
1	GAPDH	Forward: AGGAGAGTGTTTCCTCGTCCC	199	63
		Reverse: TGCCGTTGAATTTGCCGTGA		
2	Claudin-5	Forward: CCCAGTTAAGGCACGGGTAG	126	53–63
		Reverse: GGCACCGTCGGATCATAGAA		
3	Occludin	Forward: TTTCAGGTGAATGGGTCACCG	242	63
		Reverse: ACTTTCAAAAGGCCTCACGGA		

**Table 2:** A summary of U-87CM effects on brain endothelial cells (bEnd.3 cells). CM: conditional media.

	The Effects of U-87CM Paracrine Factors	
Endothelial Parameters	Normoxia (21% O_2_)	Hypoxia (5% O_2_)
**Mitochondrial dehydrogenase**	Suppressed after 72 and 96 h.	Suppressed after 24 h, 72 and 96 h.
**Mitochondrial membrane potential**	Depolarization after 24 h.	Hyperpolarization after 48, 72, and 96 h exposure to the 75% U-87CM.
**ATP production**	Decrease after 72 and 96 h.	Decrease after 48 h at 75% concentration, and after 72 and 96 h exposure.
**TEER**	Decrease after 24 h coculture, then recover to TEER control. Decrease after 24 h exposure to U-87CM.	Decrease after 24 h coculture with U-87 cancer cells but do not recover. Decrease after 48 h exposure to U-87CM at 40% and 75%.

## Data Availability

All experimental data collected are archived within the University of the Western Cape (UWC) archives and are available as per UWC data and intellectual property policy guidelines and their associated copyright protection.
